# A High-Efficiency Wind Energy Harvester for Autonomous Embedded Systems

**DOI:** 10.3390/s16030327

**Published:** 2016-03-04

**Authors:** Davide Brunelli

**Affiliations:** Department of Industrial Engineering, University of Trento, Via Sommarive 9, Trento I-38123, Italy; davide.brunelli@unitn.it; Tel.: +39-0461-285-221

**Keywords:** energy harvesting, maximum power point tracking, micro wind turbine, renewable energy application

## Abstract

Energy harvesting is currently a hot research topic, mainly as a consequence of the increasing attractiveness of computing and sensing solutions based on small, low-power distributed embedded systems. Harvesting may enable systems to operate in a *deploy-and-forget* mode, particularly when power grid is absent and the use of rechargeable batteries is unattractive due to their limited lifetime and maintenance requirements. This paper focuses on wind flow as an energy source feasible to meet the energy needs of a small autonomous embedded system. In particular the contribution is on the electrical converter and system integration. We characterize the micro-wind turbine, we define a detailed model of its behaviour, and then we focused on a highly efficient circuit to convert wind energy into electrical energy. The optimized design features an overall volume smaller than 64 cm${}^{3}$. The core of the harvester is a high efficiency buck-boost converter which performs an optimal power point tracking. Experimental results show that the wind generator boosts efficiency over a wide range of operating conditions.

## 1. Introduction

The coming decade will see the rapid diffusion of distributed standalone embedded systems and Internet of Things IoT devices, which are required to operate unattended for several years and users should only deploy-and-forget about them. The use of rechargeable battery technology is problematic due to lifetime issues, battery self-discharge, number of recharge cycles and long-term environmental impact. Low power energy harvesting is a promising technology which aims at perpetually powering the systems by extracting and converting ambient energy into electricity. The first markets for this technology are applications where direct energy supply from the power grid is not possible (or would be strongly limiting for the application) and the periodic replacement of batteries would be too expensive, such as building and home automation, military, avionic and communication devices. Harvesting will facilitate the diffusion of Internet o things (IoT) and sensing solutions based on small, low-power embedded systems, such as the nodes of wireless sensor networks [1,2]. Several power management techniques have tackled the reduction of power consumption for embedded systems ranging from Sub-Nyquist data compression [3], to transient computing [4,5], to radio usage optimization [6]. Nevertheless, in these scenarios, relying on energy sources freely provided by the operating environment and available on the spot is highly desirable.

Depending on the operating environment and on the application scenario different energy sources and different energy conversion techniques can be adopted. For example, for wearable applications, body heat, vibrations and human moving can be appropriate for the purpose [7], whereas sunlight and wind can provide enough energy for almost all outdoor applications. Any energy source is characterized by specific features in terms of controllability, predictability and signal amplitude [8,9,10,11,12]: all these three factors are relevant for the choice of the most suited source for a certain application.

Energy density, efficiency, size and cost of the harvester are the primary design metrics to compare each solution. In fact, the optimization of the harvesting capability becomes crucial to satisfy the power needs of the embedded systems and to keep the smallest size as possible.

Another critical issue is to address the variability of environmental energy sources. Indeed, for every input energy level, the harvester should exhibit different electrical loads to maximize the generated power.

The direct connection of the harvesting device to the energy storage device often does not provide the maximum harvesting efficiency, therefore the harvesting system must continually perform a Maximum Power Point Tracking (MPPT), by adjusting the operating point (*i.e.*, the impedance measured at the harvesting device output terminals) to maximize the power generation at every working condition. The design of the MPPT circuit therefore requires the characterization of the harvesting device output impedance for different input energy levels and the choice of the technique to modify the load shown at its terminals. The MPPT algorithm, then, can be implemented in hardware, through a designed analogue circuit, or executed as a piece of software by a microcontroller or a DSP.

We present the design of a wind energy harvester, starting from the work described in [13], with an overall volume below 100 ${cm}^{3}$, suitable for supplying small-sized embedded systems, installed outdoor. It consists of a small turbine-based wind generator and a highly efficient MPPT circuit, based upon a DC/DC buck-boost converter designed for the purpose. The generated energy is stored in a supercapacitor and an additional buck-boost converter is used to provide constant DC output voltage. This harvester has been designed as a plug-in module of a multi-source energy harvester with an architecture similar to [14,15,16] with hybrid storage capability. Therefore in the proposed design, an analogue control circuitry of the harvester has been thought to be powered by a secondary battery with 3.6 V nominal voltage.

The remainder of the paper is organized as follows. In the next section we review the related works with a detailed comparison between the characteristics of the proposed method and the state of the art, whereas Section 3 lists the contributions and the innovations of the paper, with respect similar solutions. In particular, we removed some design inefficiencies and we added new features. The description of the chosen wind generator, its characterization and modeling, and its performance are reported in Section 4. The MPPT circuit is the subject of Section 5, which details the design process followed to optimize the efficiency of the buck-boost converter and presents the architecture of the control circuit. Section 6 illustrates the testing procedure of the implemented harvester and shows the experimental results thus obtained, which prove to be very adherent to those expected on the basis of computer simulations. Finally, Section 7 concludes the paper.

## 2. Related Works

Although the design of self-powered embedded systems is a recent challenge, the research activity in this area is very active and several harvesters using various environmental sources have been proposed. For example, [17] reviews the latest progress in kinetic energy harvesting for wearable and mobile applications, [18] designs integrated microgenerators exploiting MEMS devices, [19] exploits vibrational energy using switched inductors, whereas [20,21] present a compact and highly efficient photovoltaic scavenger for wireless sensor networks and embedded devices.

Small scale wind energy is still quite unexplored and only a limited number of wind-flow harvesters with a size in the order of 1 ${dm}^{3}$ has been presented in literature so far. Nevertheless research in this field can leverage on the experience in designing energy generators with large and medium turbines which is almost consolidated [22].

First prototypes were proposed in [23], where piezoelectric bimorphs elements are used to generate electricity using three horizontal-axis wind turbines with a diameter of 12.7 cm each. Although they partially overlap, the whole system occupies a volume greater than 25 cm × 18 cm × 7 cm. The authors state that it can generate continuously 5 mW with an average wind speed of 16.1 km·h${}^{-1}$, and that the cut-in speed of the windmill is of about 8.7 km·h${}^{-1}$. The energy density in [23] is lower in comparison with the results presented in this work. Piezoelectricity is generally a well explored technology for converting energy from air flow. Most of the effort in improving the performance is on the particular design of the mechanics and the cantilever. Nevertheless, despite the high efficiency obtained, the size of such solutions is still too large for compact standalone embedded systems, as reported in several recent papers [24,25,26]. For example, a MEMS wind harvester based on piezoelectric element which produces maximum 16 μW output power at 15.9 m/s wind speed is presented in [27] and improves a version presented in [28].

The authors of [29] try instead to extend the battery life in a wind speed sensing application exploiting the same cup anemometer used to perform the measurements, connecting to its shaft an axial-flux brushless generator. The two round plates making up the latter have a diameter of 7.6 cm, while the cup anemometer is probably larger. Also at high speed winds (more than 30 km·h${}^{-1}$) the generated power remains below 1 mW. This poor *power/speed ratio* is mainly due to the low efficiency of the cup anemometer as a wind generator.

Ambimax [30] is a multi-source energy harvester equipped with a wind energy harvesting subsystem. This horizontal-axis turbine-based generator has a size of 38.5 cm × 32 cm × 21 cm and is capable to generate 0.5 W at 2000 rpm; from the paper it can be deduced that it is expected to generate 200 mW with a wind speed of 36 km·h${}^{-1}$. This considerable power level is probably due to the large area swept by the relatively long blades and to the favorable test conditions. However, the optimization of the efficiency of the wind energy harvesting subsystem is not thoroughly addressed in this work.

Another multi-source harvesting solution featuring a small-sized wind generator are the ones presented in [31,32]. In particular, the system described in [32] exploits a Savonius wind turbine with a swept area of 120 $cm^{2}$, capable of generating about 3.6 mW at a wind speed of 16.8 km·h${}^{-1}$, in MPP conditions. While the multi-source harvester proposed in [31] exploits a particular hybrid rectifier presented in [33], which improves the performance at low-speed of the wind and thus it is suitable for tunnels and other indoor environments.

An interesting commercial wind harvesting solution is microWindbelt [34] by Humdinger. This wind generator bases its functioning on the aeroelastic flutter principle, instead of using a turbine: this allows the whole system to be very small, with a size of just 13 cm × 3 cm × 2.5 cm. At the generator terminals, before the AC/DC power conditioning stage, power levels of 0.2 mW and 2 mW have been measured with wind speeds of 12.6 km·h${}^{-1}$ and 19.8 km·h${}^{-1}$, respectively. Although the losses due to the following power stage are not included, these power levels are rather relevant though, if we take into account the very small size of the device. The cut-in speed of microWindbelt is 10.8 km·h${}^{-1}$.

Finally the most recent implementations are from Wu *et al.* [35], and Rezaei-Hosseinabadi *et al.* [36] which use adaptive MPTT and piezoelectric beams, respectively. These two technologies permit to increase a bit the efficiency of conversion, despite the increment of the size and of the cost of the harvester. The most mature and challenging design using micro wind turbine we reviewed and compared has been proposed by [37]. They exploit a microcontroller to perform an accurate and complex maximum power point tracking, which needs the continuous measurements of both the current and the output voltage provided by the turbine. An ultra low power PWM generator is used to adjust the control signal of a boost converter. The authors achieve an electrical efficiency which is close to 60% which is remarkable.

Table 1 compares the characteristics of all the wind energy harvesters available in the literature. It lists for each system the size, the capability to track the maximum power point if it change with the wind speed, the minimum wind speed required to obtain intake energy for the storages and performance about power capability normalized over the size of the harvester and the wind speed. Not all the metrics are available from the related papers and most of them are not provided under equal environmental condition.

With a specific regard to the design methodology, there are several works dealing with the problem of maximizing the power generated by a harvesting device through the optimization of each stage of the circuit. Modeling the energy generator is one of the most difficult steps and it is important for any kind of environmental energy transducer and for high-level system simulators (e.g., WSN simulators, platfrom simulators, ...) such as the one proposed in [38]. For example, the authors of [39] achieved vibrational energy harvesting with very low power dissipation starting from a small-deflection model of the vibrating piezoelectric cantilever.

In the same way, the article [40] presented a compact and accurate model of small-size photovoltaic cells to maximize the efficiency of solar harvesters for self-powered systems. The model allowed the authors to propose a design methodology for scavenging circuits, which has been supported also by the work presented in [41].

Maximum power point trackers and DC/DC circuits are also very important optimization targets. Much effort has been invested in dynamically matching the impedance at the generator output, to maximize the energy converted with minimum energy loss. The work presented in [42] has been one of the firsts to address this issue. It proposes to implement the MPPT through a DC/DC switching converter operating in fixed-frequency discontinuous current mode (FF DCM), and a control circuit capable of varying some of the converter parameters. In this way it is possible to adjust the converter input resistance, setting its value as a function of the power level generated by the harvesting device. Whereas in this work the authors use a DSP-based power greedy control circuit to track the MPP and change the input resistance, in a following paper [43] they manage to obtain the same functionalities with a lower-consumption dedicated analogue circuit.

In some favorable cases, the output resistance of the harvesting device does not change significantly when the environmental power level varies, so a fixed load resistance is sufficient to achieve the impedance matching in every condition. This enables an additional simplification of the converter control circuit, which does not have to track the MPP and requires one-time calibration. This situation characterizes works [44,45,46,47], and will be encountered in the present paper as well.

In particular, [44] contains a brief survey of the suitability of different converter topologies (buck-boost, boost, and buck) to achieve the desired matching. This work highlights how the boost (or buck) converter operating in FF DCM is valuable only when the input voltage is much smaller (larger) than the output voltage, whereas the buck-boost converter can provide true resistor emulation independently of the input and output voltage. Therefore, the choice of the most suitable converter topology depends on the voltage characteristic of the particular application. In [45,47], e.g., a buck-boost converter is selected, whereas in [42,43] a buck converter is used. In [44] a comparison between the boost and the buck-boost topologies is also performed.

## 3. Contributions

The aim of this work is to develop a very-small-size wind energy harvester providing a highly effective solution to collect freely available wind power. To achieve this goal, the paper presents the following contributions.

- We thoroughly characterize and model the wind generator performance, to identify the conditions which maximize power generation. The selected turbine can generate up to 10 mW with a wind speed of 16 km·h${}^{-1}$, despite having a smaller size than [23,29]. The cut-in speed is lower than 8.6 km·h${}^{-1}$.
- We designed and optimized a buck-boost converter based MPPT circuit, to emulate at the wind generator output the resistive load which maximizes its performance. The adopted design methodology enhances the solution proposed by [31] and is aimed to the minimization of the power losses of the operating devices. After the selection of the components with the features most suitable to build the converter, the values of other converter parameters are chosen through extensive computer simulations and the comparison of the resulting efficiency plots. This procedure allows to make the best design choices on the basis of the expected operating conditions of the harvester.
- The architecture of the circuit controlling the converter operation is carefully designed to minimize the power consumption as well, while using COTS components. An ultra-low-power comparator is used to disconnect the oscillator circuit from the power supply when the wind is absent, avoiding the converter to continue switching when it is not needed.
- The results of the tests carried out on the implemented harvester attest the effectiveness of the applied design methodology, as they are very similar to those obtained through the numerical simulations. The measured efficiency of the converter is always greater than 81% for output voltages above 0.8 V, with peaks of 87%.

## 4. Wind Generators Characterization

With the expression *turbine-based wind generator* we identify a device capable of converting the kinetic energy of an airflow into electrical energy. We focused on the permanent-magnet version, the most suitable for low power levels. The characteristics of both the electrical generator and the mechanical turbine play an important role in determining the wind generator overall performance, in terms of efficiency, reliability and cost.

For micro-size systems like the one we present, we considered basically two types of wind turbines, namely *horizontal-axis* and *vertical-axis* generators. Horizontal-axis wind turbines (HAWT) are generally more efficient than Vertical-axis ones (VAWT), nevertheless VAWT category are simpler and can be found with several rotor designs (e.g., Darrieus, Savonius, …). Savonius rotors, for example, present some advantages over HAWTs, such as they do not need to move on the horizontal plane when the wind shifts.

**Figure 1:**
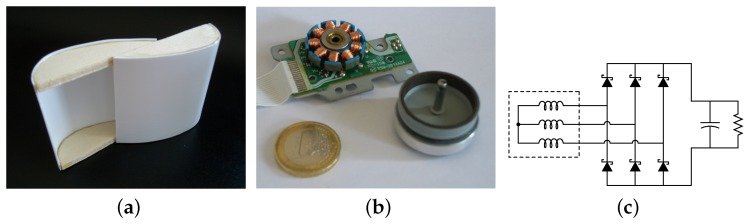
The small vertical-axis wind turbine used for the comparison and the inrunner brushless generator coupled with it. (**a**) Plastic Savonius turbine; (**b**) Disassembled motor; (**c**) Rectifier circuit.

**Figure 2:**
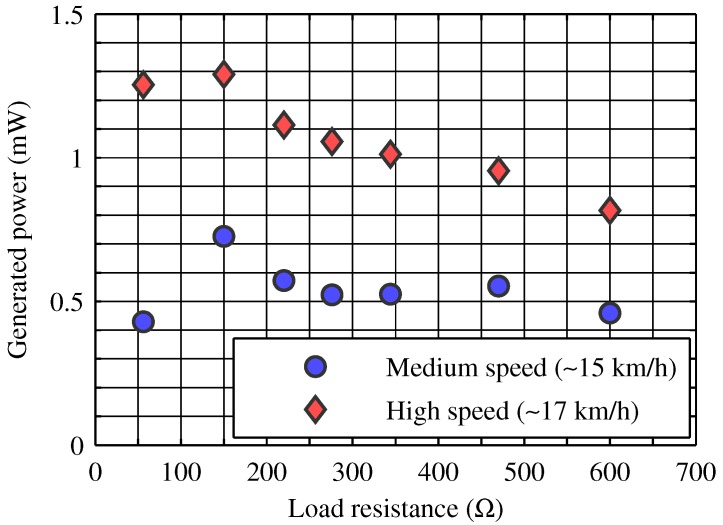
Outcome of the tests executed on a self-built vertical-axis wind turbine with a size below 100 ${cm}^{3}$. The maximum power obtained using a 17 km·h${}^{-1}$ wind has been of about 1.3 mW.

In the first instance, we investigated the suitability of small Savonius turbines (like the one displayed in Figure 1a for our purpose. We used a little three-phase motor as electrical generator Figure 1b, and a three-phase rectifier bridge with Schottky diodes to get a DC voltage from the AC waveforms Figure 1c). Then we tested their performance as wind generators at different wind flow speeds, varying several times the resistive load at the rectifier output. The results of the tests are reported in Figure 2. As the plot shows clearly, we managed to obtain an output power of 1.3 mW with an airflow speed of about 17 km·h${}^{-1}$. Moreover, the condition for the maximization of the generated power seems to be the presence of a load of about 150 Ω at the output of the rectifier.

Even though characteristics seem promising, some parameters are not still enough for large and reliable deployments, In particular VAHT still have a high cut-in speed, which is the wind speed at which the turbine starts supplying useful output power at the shaft and thus electrical power can be generated for the load.

The research has been then focused on HAWT models, in particular, after several experiments a four-blade plastic turbine with a diameter of just 6.3 cm has been selected. The generator consists of a single-phase coil, which encloses the magnets integral with the rotating shaft attached to the turbine.

**Figure 3:**
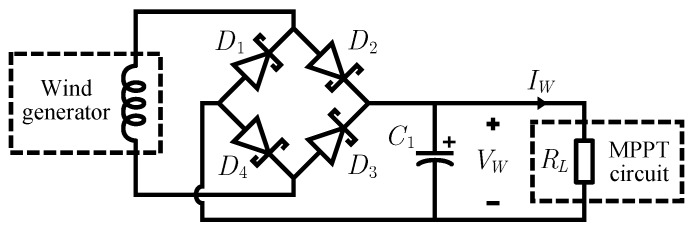
Circuit diagram of the full-wave rectifier used to convert the voltage generated by the wind generator from AC to DC.

The circuit used to convert the generated AC supply voltage to a DC voltage for the embedded system is a typical single-phase full-wave rectifier, with a diode bridge followed by a filter capacitor, as shown in Figure 3. A BAT47 Schottky diode is used to minimize voltage drop and power losses. The filter capacitor value (220 μF) is the result of a tradeoff between the minimization of the voltage ripple and the ability of the output voltage to follow closely enough the wind speed variations. This last requirement is important if the rectifier output voltage is to be used as the feedback signal for the MPPT circuit: as it should modify its operation on the basis of actual environmental energy level, a delay in the feedback chain is undesirable.

For the characterization of the wind generator we used three airflow speeds as reported in the second column of Table 2. The measurements are made at the rectifier output port and, this, the power losses caused by the diode bridge are already considered in the measurement, as well as their variations caused by different input power levels.

**Figure 4:**
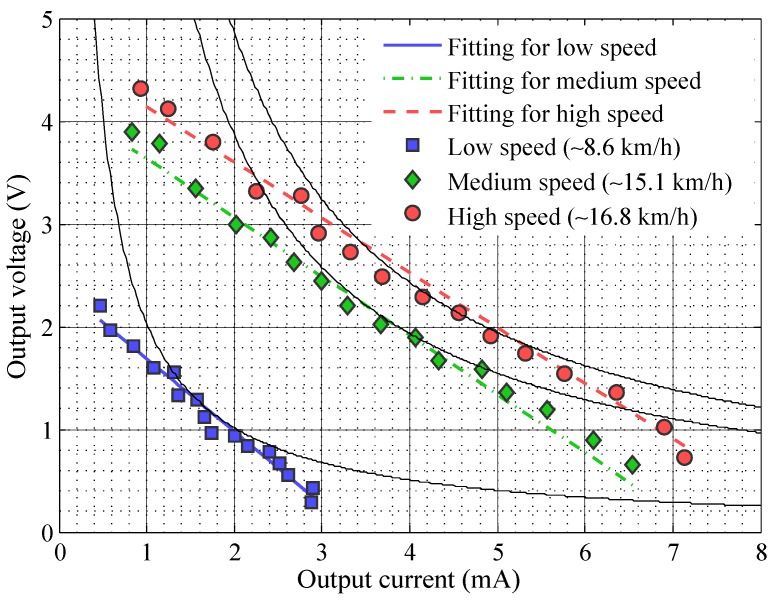
Characterization of the horizontal-axis wind turbines (HAWT) displayed on a $V_{W}$-$I_{W}$ plot, together with the fitting curves resulting from the analytic model. The solid black curves represent constant power levels: from left to right, the power levels associated to the three curves are 2.02 mW, 7.93 mW, and 9.95 mW (see Table 2).

The outcome of the measures on the HAWT model is displayed in Figure 4. This plot reports the experimental samples of the wind generator *V*-*I* characteristics, parametrized by the level of input power which depends on the airflow speed. Generally, for a fixed airflow speed, there is a load value which maximizes the power generated by the wind turbine. Notice that with an airflow speed of about 17 km·h${}^{-1}$, it is possible to generate up to about 9.7 mW: this power level is well above those obtained by [23,29]. To summarize, the HAWT model can generate up to 7 times more power with respect to the vertical one we built, using the same wind speed. Thus we continued the design of the harvester considering this model of turbine.

The rectilinear position of the three groups of data points on this plot suggests the possibility to model the generator behavior at a fixed input power level with a linear model of the following type:

(1) $$V_{W}=-p_{1}I_{W}+p_{2}\;$$

where $I_{W}$ and $V_{W}$ are the output current and voltage from the wind generator and $p_{1}$ and $p_{2}$ are fitting parameters whose values are dependent on the wind speed (input power). After performing a least mean squares fitting, we obtain the values reported in Table 2 for parameters $p_{1}$ and $p_{2}$ and the lines in Figure 4, superimposed on the experimental data points.

Now, it is possible to use the model to get a better estimate of the resistance value which optimizes the power generation at a fixed airflow speed. Knowing that the output power is given by $P_{W}=V_{W}I_{W}$ and using Equation (1), we obtain that:

(2) $$P_{W}=-p_{1}I_{W}^{2}+p_{2}I_{W}\;$$

Differentiating the previous equation it is easy to determine the current value which maximizes the output power and consequently the expression of the maximum power achievable:

(3) $$I_{W,\mathrm{opt}}=\frac{p_{2}}{2\;p_{1}}\;$$

(4) $$P_{W,\max}=P_{W}\left(I_{W,\mathrm{opt}}\right)=\frac{p_{2}^{2}}{4\;p_{1}}\;$$

Hence the load value which optimizes the power generation is:

(5) $$R_{L,\mathrm{opt}}=\frac{P_{W,\max}}{I_{W,\mathrm{opt}}^{2}}=p_{1}\;$$

Another interesting result synthetically describing the dependence of the generated power on the ambient energy level (through $p_{1}$ and $p_{2}$ values) and the load resistance can be found putting Equation (2) in a system with $P_{W}=R_{L}\;I_{W}^{2}$:

(6) $$P_{W}=\frac{R_{L}\;p_{2}^{2}}{{\left(R_{L}+p_{1}\right)}^{2}}\;$$

Table 2 reports the values of $P_{W,\max}$ and $R_{L,\mathrm{opt}}$ corresponding to the three wind flow speeds used to characterize the turbine. It is immediate to observe that the optimal load resistance value lies between 549 Ω and 715 Ω for all the tested airflow speeds. The narrowness of this range implies that, if a fixed load resistance value is chosen within this range, the power generated for any input power level will be close to the maximum one.

The choice of the exact value for the load resistance is based on which operating condition one wants to optimize. The trend noticeable in Table 2 is a slight decrease in $R_{L,\mathrm{opt}}$ when the airflow speed increases: considering that our characterization stops at an airflow speed corresponding to a “gentle breeze” in the Beaufort scale [48], we expect the $R_{L,\mathrm{opt}}$ value to further decrease at higher wind speeds. Our tradeoff choice is then a resistance value of 550 Ω for the load of the rectifier: it allows the generation of the maximum power with low speed air flows (which are also the most frequent ones) without penalizing too much the efficiency at higher wind speeds.

## 5. MPPT Circuit

Considering the model of the wind turbine used in our harvester, the condition which maximizes the power supplied by the wind generator (rectifier included) is approximated assuming the presence of a fixed load resistance of about 550 Ω. The transfer of the energy to the selected storage device is independent on the level of energy already stored into it.

Note that in such case there is no real “tracking” of the maximum power point, because the conditions for the maximization of the generated power are only slightly dependent on the input power level. This particularity brings some advantages. In the first place, the absence of the feedback control circuitry reduces the system complexity and the implementation costs. Moreover, this means also no additional power consumption. If a more sophisticated MPPT circuit, able to precisely modify its input resistance in response to operating conditions variations, was employed, it would consume more power than that gained thanks to its more accurate form of tracking (with respect to the approximate condition we have chosen). Indeed, even if we consider the case of worst mismatch between $R_{L,\mathrm{opt}}$ and the chosen value of 500 Ω (happening with the lowest airflow speed, see Table 2), the power lost because of the mismatch is just the 2% of the $P_{W,\max}$ value, quantifiable in 40 μW. The power consumption of a more sophisticated MPPT circuit would hardly be below this power level.

In Section 2 we reviewed some works dealing with the emulation of a constant resistance. They all adopt a DC/DC converter operated in fixed-frequency discontinuous current mode (FF DCM), and this solution seems suitable to our case as well. In our context, both input and output voltages can reach about the same level. The converter input voltage is the output voltage of the rectifier, which can reach several volts as soon as the wind strengthens, whereas the converter output voltage is the voltage on the supercapacitor, which can range from 0 V to 5 V according to the amount of energy already stored. For this reason, we have chosen to employ a buck-boost converter operated in FF DCM to satisfy the condition for the maximization of the power supplied by the wind generator.

### 5.1. Buck-Boost Converter

**Figure 5:**
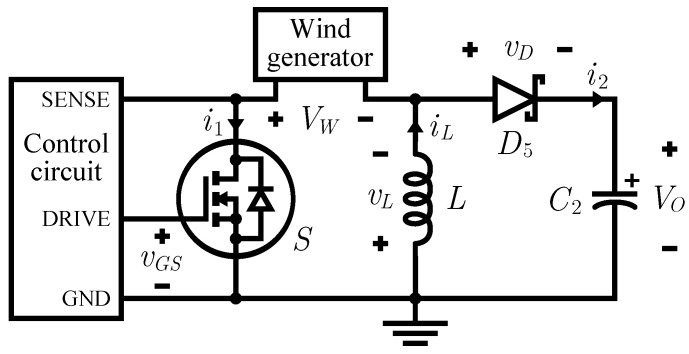
Circuit diagram of the considered buck-boost converter.

**Figure 6:**
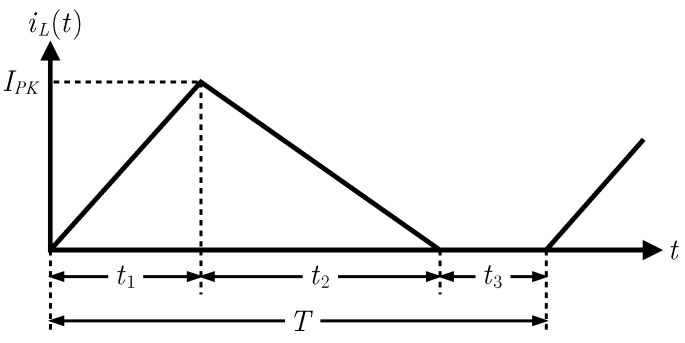
Waveform of the current through the inductance L, when the DC-DC is operating in fixed-frequency discontinuous current mode.

The circuit diagram of the considered buck-boost converter is shown in Figure 5. The qualitative waveform of the current through the inductance *L* of the converter operating in FF DCM is reported in Figure 6. For a thorough investigation of converter operation in this mode, see [49].

Referring to Figure 6, $t_{1}$ is the time interval in which the MOSFET is in conduction, $t_{2}$ is the time it takes for the inductor to transfer all the energy stored during $t_{1}$ to the supercapacitor while the MOSFET is off and $t_{3}$ is the remaining time before the start of the next period. The overall period length is denoted by *T*.

Modeling the MOSFET and the diode as ideal switches and integrating the basic relation $v_{L}\;=\;L\;\frac{di_{L}}{dt}$ regarding the converter inductor, it is possible to obtain the following expression for the peak current in the inductor:

(7) $$I_{\mathrm{PK}}=\frac{V_{W}}{L}t_{1}=\frac{V_{O}}{L}t_{2}\;$$

The waveform of the current $i_{1}$, drawn from the rectifier at the end of the wind generator, is equal to that of $i_{L}$ during $t_{1}$, but it is zero during the intervals $t_{2}$ and $t_{3}$. The expression of its average value is then:

(8) $$I_{1,\mathrm{avg}}={\langle i_{1}\left(t\right)\rangle }_{T}=\frac{1}{T}\int_{t_{0}}^{t_{0}+T}i_{1}\left(\tau \right)\;d\tau =\frac{t_{1}I_{\mathrm{PK}}}{2\;T}\;$$

The average power entering the converter is given by $P_{\mathrm{IN},\mathrm{avg}}=V_{W}I_{1,\mathrm{avg}}$.

From Equations (7) and (8), this formula can be rewritten as follows:

(9) $$P_{\mathrm{IN},\mathrm{avg}}=V_{W}\left[\frac{t_{1}}{2\;T}\left(\frac{V_{W}}{L}t_{1}\right)\right]=V_{W}^{2}\left(\frac{t_{1}^{2}}{2\;L\;T}\right)\;$$

Indicating with $R_{\mathrm{IN},\mathrm{eq}}$ the equivalent input resistance of the converter, this quantity verifies the equation:

(10) $$P_{\mathrm{IN},\mathrm{avg}}=\frac{V_{W}^{2}}{R_{\mathrm{IN},\mathrm{eq}}}\;$$

from the comparison of this expression with Equation (9), it is immediate to obtain:

(11) $$R_{\mathrm{IN},\mathrm{eq}}=\frac{2\;L\;T}{t_{1}^{2}}\;$$

which demonstrates that the buck-boost converter operating in FF DCM shows a fixed average input equivalent resistance, dependent only on the inductance value and on two time parameters which characterize the waveform of the signal to drive the gate, as reported by textbooks on power electronics [50].

#### 5.1.1. Designing for Maximum Efficiency

The possibility of choosing the values of *L*, $t_{1}$, and *T* permits to have three degrees of freedom.

Thus, it is fundamental to provide some guidelines for optimizing the design of the harvester circuit for achieving the maximum efficiency. Usually, optimizing the harvester circuit efficiency requires iterative elaborations and simulations, as demonstrated in [51]. The principal focus during the design is reducing the losses due to parasitic resistance, to the MOSFET switching activities and to the inductor. Generally, these power losses depend on the maximum inductor current and the operating frequency, as well as on MOSFET parasitic capacitance. The design process requires an iterative procedure to select the component values that maximize the circuit efficiency. In the following, we discuss show how we organized the simulations to draw conclusions about the component selection.

First of all, considering that there is only the following constraint to satisfy:

(12) $$R_{\mathrm{IN},\mathrm{eq}}=R_{L,\mathrm{opt}}=550\;\Omega \;$$

two parameters can be arbitrarily set by the designer for achieving the maximum efficiency. A good way to take advantage of this possibility is to choose for *L*, $t_{1}$, and *T* the values which minimize the power losses due to the components including the converter, thus maximizing its efficiency. This approach is used also by [44].

To evaluate the conduction losses of the MOSFET, the inductor, and the diode, and the switching losses of the MOSFET, the following equations can be respectively used:

(13) $$\begin{array}{cc} P_{S,\mathrm{cond}} & =R_{\mathrm{on}}\;I_{1,\mathrm{rms}}^{2}=R_{\mathrm{on}}\;I_{\mathrm{PK}}^{2}\left(\frac{t_{1}}{3\;T}\right)\\ P_{L,\mathrm{cond}} & =R_{\mathrm{esr}}\;I_{L,\mathrm{rms}}^{2}=R_{\mathrm{esr}}\;I_{\mathrm{PK}}^{2}\left(\frac{t_{1}+t_{2}}{3\;T}\right)\\ P_{D,\mathrm{cond}} & =\frac{n\;V_{t}\;L\;I_{\mathrm{PK}}^{2}}{2\;T\;V_{O}}\left[\ln\left(\frac{I_{\mathrm{PK}}}{I_{S}}\right)-\frac{1}{2}\right]\\ P_{S,sw} & =\frac{1}{T}\;C_{\mathrm{oss}}\frac{V_{W}^{2}}{2} \end{array}$$

where $R_{\mathrm{on}}$ is the drain-source on-state resistance of the N-MOSFET, *n* is the ideal factor of the diode, $R_{\mathrm{esr}}$ is thbe parasitic equivalent series resistance of the inductor, $I_{S}$ is the reverse bias saturation current of the diode, $V_{t}$ is the thermal voltage, and $C_{\mathrm{oss}}$ is the MOSFET output capacitance. The expression indicated for $P_{D,\mathrm{cond}}$ has been obtained considering the waveform of the current through the diode (equal to that of $i_{L}$ in Figure 6 during the time interval $t_{2}$) and the waveform of the forward voltage after the classic Shockley equation $v_{D}\left(t\right)=n\;V_{t}\ln\left(\frac{i_{D}\left(t\right)}{I_{S}}+1\right)$.

Note that in the expression of the MOSFET switching losses $P_{S,\mathrm{sw}}$, the power consumed by the MOSFET driving circuit to charge the gate capacitance at every cycle has not been included. Indeed this power is provided by the secondary battery and not by the converter input port, so it is not relevant to the considered conversion efficiency.

As Equation (13) show, the losses depend on the three parameters. Hence, also the devices must be selected carefully to maximize the efficiency. The inductor should have the smallest possible $R_{\mathrm{esr}}$: this requires a thicker conductor, which makes the inductor quite larger. The MOSFET should have both a low $R_{\mathrm{on}}$ and a low $C_{\mathrm{oss}}$: a tradoff between the two is mandatory, as lower $R_{\mathrm{on}}$ can be achieved by widening the transistor, therefore increasing the parasitic capacitances and the gate charge $Q_{g}$. The diode should have the smallest possible forward voltage at the $I_{\mathrm{PK}}$ current level: this is often associated with higher reverse bias leakage currents, which is undesirable.

After a thorough comparative research, we selected the components listed in Table 3. Both the MOSFET and the Schottky diode are integrated in the same package, the NTMD4884NF by On Semiconductor. Note that at this design stage it is not possible to choose a particular inductor only on the basis of its $R_{\mathrm{esr}}$: in fact the $R_{\mathrm{esr}}$ value of an inductor is strictly related to its inductance value, so the choice of the inductor must consider both the features at the same time. The next paragraph will explain how this can be accomplished.

#### 5.1.2. Simulation

The best values for the parameters $t_{1}$, *L* and *T* has been performed through computer simulations. Equation (13) have been evaluated with MATLAB, obtaining the total power loss, which has been calculated using many different couples of values to parameters *L* and $t_{1}$. The value of *T* has been calculated from the condition summarized by Equation (12), using Equation (11). Many simulations have been carried out varying the input power level and the voltage considered for the output supercapacitor. This allowed to evaluate the efficiency performance of the converter for the widest possible range of operating conditions. At each change of the inductance value *L*, also the $R_{\mathrm{esr}}$ value has been modified according to a table containing the *L*–$R_{\mathrm{esr}}$ correspondences for a wide selection of the best inductor families available on the market.

**Figure 7:**
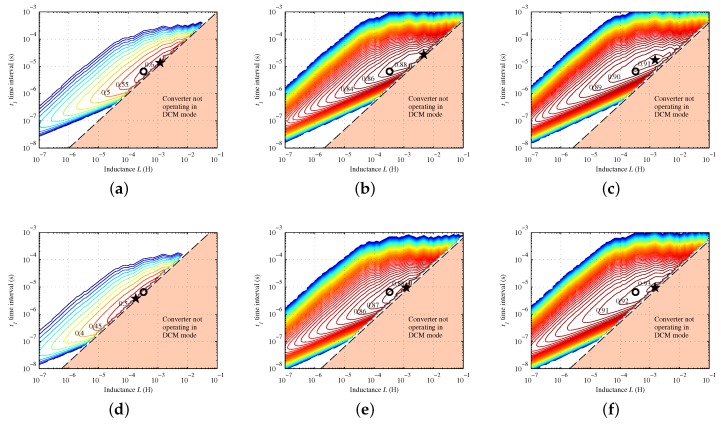
Simulation results of the efficiency performance of the converter for the a wide range of operating conditions ($V_{O}$ and $P_{\mathrm{IN}}$). (**a**) (0.5 V, 2.0 mW); (**b**) (2.0 V, 2.0 mW); (**c**) (3.5 V, 2.0 mW); (**d**) (0.5 V, 9.8 mW); (**e**) (2.0 V, 9.8 mW); (**f**) (3.5 V, 9.8 mW). The maximum efficiency point is denoted by the star symbol. The efficiency achieved by the final implementation is denoted by a circle.

The result of some of these simulations are shown in Figure 7. Notice that the plot stops abruptly on the right side: this is because the values of *L* and $t_{1}$ corresponding to the points in the lower rightmost triangular area of the plot would not allow the converter to operate in DCM, making $t_{1}\;+\;t_{2}\;>\;T$.

Changing the operating conditions ($V_{O}$ and $P_{\mathrm{IN}}$), the borderline between DCM and non-DCM operation moves, as does the maximum efficiency point, denoted by the star symbol. In any case, however, the maximum efficiency point is always quite near to the borderline. Therefore, the choice of the best values for *L* and $t_{1}$ should correspond to a point as near as possible to the area where the maximum efficiency point lies more often, but still remaining on the left of the borderline for the widest possible range of operating conditions. In general, the risk of crossing the borderline is higher in presence of great input power levels and a low voltage across the output supercapacitor.

After the comparison of a large set of plots obtained from the simulation of several different operating conditions, the tradeoff values of 330 μH and about 6.5 μs have been chosen for *L* and $t_{1}$, respectively. To satisfy the condition of Equation (12), a period T=35.2μs is required, corresponding to f=1/T=28.4kHz. The selected inductor is the ELC11D331F by Panasonic, which features an $R_{\mathrm{esr}}$ of only 350 mΩ.

**Figure 8:**
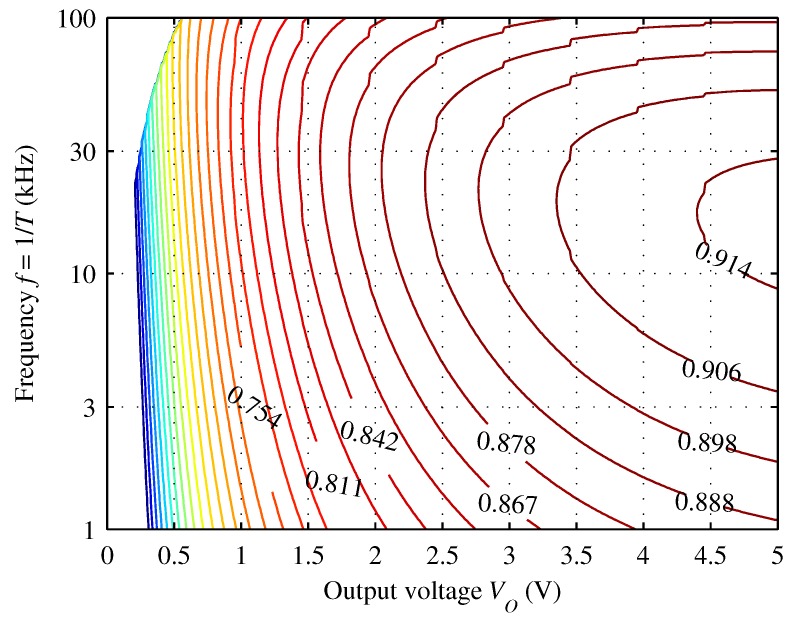
Contour plot of the conversion efficiency corresponding to different values of *f* and $V_{O}$, once L=330μH has been chosen. For this simulation we supposed $P_{\mathrm{IN}}=2.0\;mW$, obtainable with the lowest speed used for our tests and with $R_{L}=R_{\mathrm{IN},\mathrm{eq}}=550\;\Omega$.

Once the circuit has been physically implemented, only the adjustment of parameters *T* and $t_{1}$ remains possible, because *L* is fixed. Since, just one of the three degrees of freedom initially available remains, it is actually possible to modify arbitrarily just one of the two parameters *T* and $t_{1}$, because the other must be tuned accordingly to Equation (12). Supposing to modify the value of *T* while adjusting accordingly $t_{1}$, it is possible to obtain the plot shown in Figure 8 by the use of computer simulation. It shows how the estimated conversion efficiency depends on the operating frequency and the voltage across the supercapacitor, assuming again the lowest airflow speed. Checking the position of f=28.4kHz on this plot, we can observe that it is among the frequencies which maximize the efficiency-increase-to-voltage-increase ratio: this means that this choice of *f* allows on average to reach better efficiencies at lower output voltages.

Picturing the plot of Figure 8 in 3D and slicing it by a fixed frequency value, we would obtain a 2D plot showing the conversion efficiency as a function of the output voltage, for a given input power level and operating frequency. Considering an *f* value around 30 kHz, we can see that the simulated conversion efficiency is greater than 75% already at $V_{O}=1\;V$; it exceeds 90% at about $V_{O}=3\;V$.

### 5.2. Control Circuit

The converter requires a circuit driving the gate of the MOSFET with a voltage square wave of period *T* and duty cycle equal to $t_{1}/T$. To maximize the harvester overall efficiency, this circuit should consume the least possible power. Besides an accurate design of the circuit itself, an architectural-level strategy to cut down its power consumption is to cut its power supply every time that the speed of the airflow is not high enough to make the turbine turn, *i.e.*, during wind calms.

The main problem arising in the implementation of this solution is how to sense the voltage at the output of the rectifier, which signals clearly whether the turbine is spinning or not. As Figure 5 shows, indeed, the voltage $V_{W}$ is *floating*, *i.e.*, none of the two electric potentials to which it is referred is the ground one. For this reason it is not possible to use a comparator powered by a ground-referred supply to directly compare this voltage to a threshold.

A simple and clever solution to this difficulty comes from a feature of the circuit in Figure 5. Applying the Kirchhoff’s voltage law to the mesh including the MOSFET, the inductor and the rectifier output, we obtain that:

(14) $$v_{\mathrm{DS}}+v_{L}=V_{W}$$

where $v_{\mathrm{DS}}$ is the drain to source voltage of the MOSFET. The first two quantities are denoted lowercase because they vary during each cycle of the converter, whereas $V_{W}$ remains practically constant over consecutive cycles, on the short term. If we average the previous equation on a period *T*, we get:

(15) $${\langle v_{\mathrm{DS}}\rangle }_{T}+{\langle v_{L}\rangle }_{T}={\langle V_{W}\rangle }_{T}=V_{W}$$

Remembering that in presence of periodic waveforms the average voltage across an inductor is zero, we obtain the following result:

(16) $$V_{\mathrm{DS},\mathrm{avg}}={\langle v_{\mathrm{DS}}\rangle }_{T}=\frac{1}{T}\int_{t_{0}}^{t_{0}+T}v_{\mathrm{DS}}\left(\tau \right)\;d\tau =V_{W}$$

It is thus possible to measure $V_{W}$ performing an average of the drain-source voltage. The simplest way to implement this operation is through a low-pass filter connected between the MOSFET drain and the ground. The cutoff frequency of the filter must be quite smaller than the switching frequency of the converter (of the order of 10 kHz), but high enough to allow the filter output voltage to follow the airflow speed variations (around some hertz) without a substantial delay. A value in the range 10 Hz to 100 Hz should be suitable for the cutoff frequency of this filter.

**Figure 9:**
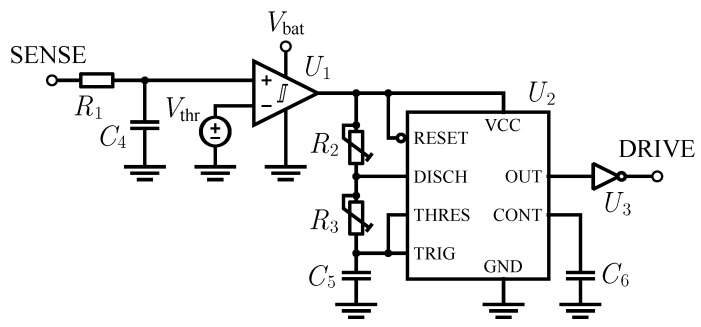
Qualitative diagram of the circuit which controls the operation of the buck-boost converter.

This is exactly the function of the $R_{1}$-$C_{4}$ network positioned at the beginning of the control circuit we designed, shown in Figure 9. As Figure 5 displays, the SENSE input is connected to the drain of the MOSFET. Through this filtering stage, the voltage $V_{W}$ is reproduced at the non-inverting input of $U_{1}$. The output of $U_{1}$ becomes high when $V_{W}$ is greater than a threshold voltage $V_{\mathrm{thf}}$ of about 200 mV; this value is high enough to protect against unwanted noise-induced commutations. For the integrated circuit $U_{1}$ we used an ultra-low power comparator with integrated reference voltage, the LTC1440 by Linear Technology. It consumes less than 3.7 μA over its full temperature operating range, but its output stage is capable of sourcing up to 40 mA. For these reasons it is the ideal device to monitor the presence of the wind and to power accordingly the oscillator stage which drives the MOSFET gate terminal.

The oscillator circuit is schematically represented on the right side of Figure 9. From a functional point of view, it is based on a 555 timer connected for astable operation. The frequency and the duty cycle of the square wave generated at the output port are determined by the values of $R_{2}$, $R_{3}$, and $C_{5}$ through the equations reported in the datasheet.Choosing a convenient fixed value for $C_{5}$, the possibility of setting just the $R_{2}$ and $R_{3}$ values is sufficient to achieve the desired range of frequencies and duty cycles. In the implementation, we used for $U_{2}$ an ICM7555 by Intersil.

Overall, the only device of the control circuit independently powered is the comparator $U_{1}$. It receives the needed energy from the secondary battery belonging to the architecture of the multi-source energy harvester which includes the wind energy harvester presented in this paper.

## 6. Experimental Results

**Figure 10:**
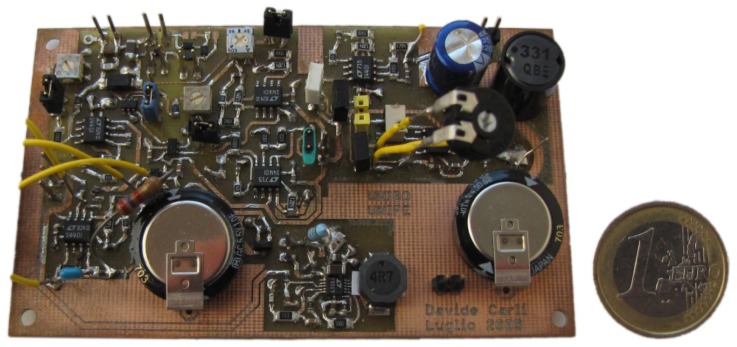
Picture of the assembled multi-source energy harvester. The wind energy harvesting section is on the rightmost side: from top downwards, the radial electrolytic capacitor $C_{1}$, the radial inductor *L*, the trimmer $R_{3}$ and the supercapacitor $C_{2}$ are clearly recognizable.

The assembled multi-source energy harvester is displayed in Figure 10. The whole PCB area is 6.7 cm × 4.7 cm. After checking the smooth functioning of all the present sub-circuits, we carried out some tests to evaluate the real performance of the designed MPPT circuit and thus to verify the accuracy of the expectations provided by the simulations.

The aim of these tests is to measure the efficiency *η* of the converter for different values of its output voltage $V_{O}$, *i.e.*, the voltage across the supercapacitor $C_{2}$. To obtain these data, we start discharging completely $C_{2}$. Then we apply at the input port of the converter a fixed voltage $V_{W,1}$, provided by a DC power supply previously set to simulate the presence of the wind generator hit by the airflow. In this way the converter begins its operation and $C_{2}$ starts charging. Afterwards, at known time instants, we measure the output voltage.

We use a DC power supply connected to the rectifier input instead of the real wind generator to avoid shifts of the operating conditions due to the turbulence of a fan-generated airflow, which would alter the experiment results. The DC voltage of the power supply has been set to obtain at the rectifier output, loaded with $R_{L,\mathrm{opt}}$, the voltage:

(17) $$V_{W,1}=\frac{R_{L,\mathrm{opt}}\;p_{2,1}}{R_{L,\mathrm{opt}}+p_{1,1}}=1.04\;V\;$$

the previous equation can be found using Equation (1) together with $V_{W}=R_{L}\;I_{W}$. $p_{1,1}$ and $p_{2,1}$ are the values of $p_{1}$ and $p_{2}$ corresponding to the lower speed used in our tests (see Table 2).

From the knowledge of the voltage $V_{O}$ at two time instants $t_{0}$ and $t_{1}=t_{0}+\Delta t$ (so that $t_{1}>t_{0}$), and using the well-known relation $E_{C}\left(t\right)=\left(1/2\right)\;C\;v_{C}^{2}\left(t\right)$ which expresses the energy stored in a capacitor of capacity *C*, it is possible to determine the average power supplied by the converter to $C_{2}$ during Δt through the following equation:

(18) $$P_{O}\left(t^{*};V_{O}^{*}\right)=\frac{\Delta E_{C}}{\Delta t}=\frac{C_{2}}{2\;\Delta t}\left[V_{O}^{2}\left(t_{1}\right)-V_{O}^{2}\left(t_{0}\right)\right]\;$$

With $t^{*}$ and $V_{O}^{*}$ we denote the time instant or the output voltage to which one wants to relate this specific value of $P_{O}$: e.g., $t^{*}$ could be assumed equal to $(t_{1}+t_{0})/2$, but also to $t_{1}$ or $t_{0}$, on the basis of the convention adopted. We were interested in the $\eta (V_{O})$ relationship, so we linked each time the average output power to the voltage $V_{O}^{*}=\left[V_{O}\left(t_{1}\right)+V_{O}\left(t_{0}\right)\right]/2$.

Once $P_{O}\left(V_{O}^{*}\right)$ has been determined, it is possible to obtain the efficiency of the converter for output voltages around $V_{O}^{*}$ using the equation:

(19) $$\eta \left(V_{O}^{*}\right)=\frac{P_{O}\left(V_{O}^{*}\right)}{P_{\mathrm{IN}}}\;$$

where $P_{\mathrm{IN}}$ is calculated as $R_{\mathrm{IN},\mathrm{eq}}\;V_{W,1}^{2}$.

Through the calculation of $\eta (V_{O}^{*})$ for some $V_{O}^{*}$ values, corresponding to consecutive time intervals, it is possible to obtain a sampling of the real $\eta (V_{O})$ curve. In theory, a more frequent measure of $V_{O}$ should permit a better approximation of the $\eta (V_{O})$ curve, but in practice this can lead to a more jagged progression of the data due to transitional deviations in the converter operation and in the supercapacitor behavior.

**Figure 11:**
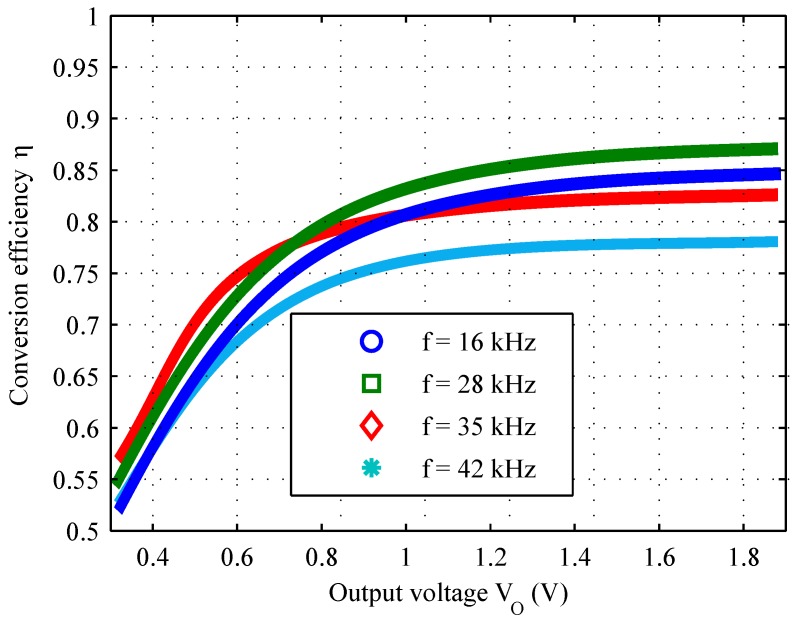
Efficiency as a function of the measured output voltage, considering different values of the switching frequency, driving the implemented converter.

We executed several tests. In particular we changed each time the switching frequency of the converter, and adjusting accordingly $t_{1}$ to satisfy Equation (12), to verify the converter performance forecast by Figure 8. The results of some of these experiments are summarized by Figure 11. Notice that, when $C_{2}$ is less charged, the efficiency is lower but it rises more quickly when $V_{O}$ is above 1 V.

Despite this slight mismatch between simulated and experimental results, the efficiency with f=28kHz is adequate and sufficient for the whole tested $V_{O}$ range: it is always above 81% when $V_{O}$ is higher than 0.8 V, with a maximum value of 87%, which is very near to that expected from the simulations.

This data are confirmed by infield experiments. With an average wind speed of 16 km·h${}^{-1}$, the harvester can continuously generate an average power of 8.3 mW, with a conversion efficiency around 84%.

If we consider the whole wind harvester, the efficiency of converting the kinetic energy of the wind into electrical energy is much less. According to the Betz’s law, no mechanic turbine can capture more than 59.3% of the wind kinetic energy, which is further reduced when conveterted into electrical energy by the efficiency of the electric converter. Measuring the overall efficiency of the system is not easy for such a small prototype. Preliminary tests, done using a test bench with controlled air flow speed, exhibit an overall efficiency of 26% including the Betz’s law contribution, as average of different measurements done with different wind speeds.

## 7. Conclusions

A highly efficient energy harvester which exploits a micro wind turbine has been proposed. It outperforms the ones proposed by similar works: it can provide up to 10 mW with an airflow speed of about 16 km·h${}^{-1}$, despite a turbine diameter of just 6.3 cm. A detailed design methodology, aimed at the minimization of its power losses, has been presented to achieve a fully analogue, highly efficient, very-small-scale wind scavenger. The plots resulting from the numerical simulations are validated through experimental tests executed on the implemented harvester. Considering the expected operating conditions, designers can be aided by simulation results to evaluate all the relevant tradeoffs and to tailor the harvester to the specific application requirements.

## Figures and Tables

**Table 1:** Comparison of the characteristics of the wind energy harvester presented the literature.

Harvester	Size	Power Density	Cut-in Speed	Power/Speed Ratio ${}^{1}$	MPPT
	(Approx. cm${}^{3}$)	(μW·cm${}^{-3}$)	(km·h${}^{-1}$)	(mW/(km/h))	Capability
Myers *et al.* [23]	3150	1.6	8.7	0.311	No
Weimer *et al.* [29]	440	2.3	N.A.	0.033	No
Park *et al.* [30]	5300	18	>12	0.55	No
Morais *et al.* [32]	1200	30	N.A.	0.21	No
Hummingbird [34]	97	21	10.8	0.101	No
Tan *et al.* [37]	125	63	8.3	0.156	Yes
Porcarelli *et al.* [33]	120	71	8.0	0.76	Yes
Xiang *et al.* [25]	90	71	19.8	0.033	No
Zhang *et al.* [24]	50	12.6	N.A.	N.A.	No
Wu *et al.* [35]	392	33.6	13.7	N.A.	Yes
Rezaei *et al.* [36]	432	2.64	7.7	N.A.	No
**This Harvester**	54	83	7.9	0.59	Yes

${}^{1}$ values extracted from the respective papers at not normalized wind speeds.

**Table 2:** Data regarding the characterization and modeling of the wind turbine used for our harvester.

Cases	Airflow Speed	$P_{W,\max}$	$p_{1}=R_{L,\mathrm{opt}}$	$p_{2}$
	(km·h${}^{-1}$)	(mW)	($VA^{-1}$ = Ω)	(V)
Low speed	8.6	2.02	715	2.40
Medium speed	15.1	7.93	559	4.21
High speed	16.8	9.95	549	4.68

**Table 3:** List of the relevant features of the components selected to implement the buck-boost converter.

Component	Features
n-channel MOSFET	$R_{\mathrm{on}}=65\;m\Omega$ at $V_{\mathrm{GS}}=3.6\;V$, $I_{D}=2\;A$
	$Q_{g}=2.4\;nC$ at $V_{\mathrm{GS}}=3.6\;V$, $I_{D}=4\;A$
	$C_{\mathrm{oss}}=80\;pC$ at $V_{\mathrm{GS}}=0\;V$, $V_{\mathrm{DS}}=6\;V$
Schottky diode	$V_{D}=0.25\;V$ at $I_{D}=60\;mA$
	$I_{R}=10\;\mu A$ at $V_{R}=5\;V$
